# Association between smartphone addiction and myofascial trigger points

**DOI:** 10.1186/s12891-024-07383-4

**Published:** 2024-04-01

**Authors:** Özden Yaşarer, Emel Mete, Reyhan Kaygusuz Benli, Berivan Beril Kılıç, Halis Doğan, Zübeyir Sarı

**Affiliations:** 1 Department of Therapy and Rehabilitation, Vocational School, Istanbul Arel University, Istanbul, Turkey; 2 Department of Physiotherapy and Rehabilitation, Institute of Health Science, Marmara University, Istanbul, Turkey; 3 Department of Physiotherapy and Rehabilitation, Faculty of Health Science, Medeniyet University, Istanbul, Turkey; 4 Department of Physiotherapy and Rehabilitation, Faculty of Health Science, Demiroğlu Bilim University, Istanbul, Turkey; 5 Department of Physiotherapy and Rehabilitation, Faculty of Health Science, Istanbul Arel University, Istanbul, Turkey; 6 Department of Physiotherapy and Rehabilitation, Faculty of Health Science, Marmara University, Istanbul, Turkey

**Keywords:** Smartphone, Mobile phone addiction, Myofascial trigger point, Forward head, Addiction

## Abstract

**Background:**

The purpose of this study was to clarify the relationship between smartphone addiction and miyafascial trigger points in university students.

**Methods:**

A cross-sectional study of university students was conducted for the purpose of this study. The participants were assessed based on age, gender, dominant side, the amount of time they spent on their smartphones, the purpose of their use, and their posture. The Smartphone Addiction Scale Short Form (SAS-SF) was used to determine addictes and non-addicts. The cut-off value of SAS-SF is 31 and above for male and 33 and above for female.

**Results:**

There were 136 participants in the study. The posture score for addicts and non-addicts ones was not significantly different (*p* > 0,05), but the number of trigger points, maximal bending posture and trigger points in the right levator scapula and right cervical erector muscles were significantly higher in the smartphone addict participants (*p* < 0,05).

**Conclusions:**

Smartphone addiction in university students is associated with postural changes and trigger points in the bilateral levator scapula and right cervical erector muscles. Public health programs should be developed to raise awareness about smartphone addiction, encourage screen breaks, and emphasize physical activity and exercise regularly.

## Background

The development of communication technologies in the twentieth century has also increased the capabilities of smartphones and, thus, their use areas [1]. With smartphones spreading to large masses in a short period of time, they are deeply embedded in the daily life practices and dreams of users in Turkey as well as in other countries. The "Digital 2019 in Turkey" report of 'We are social' and 'Hootsuit' revealed that 98% of Turkish adults use smartphones, and 77% own smartphones [2].

Since smartphones are not only devices for communication but also contain features such as cameras, GPS navigation, entertainment games, and social media, particularly young individuals tend to spend more time with their smartphones [3, 4]. Overuse of smartphones can result in addiction [5]. In recent years, smartphone addiction has become a major global problem affecting people of all ages, especially university students [6, 7]. In university students, smartphone addiction has been associated with decreased academic performance, musculoskeletal pain, inadequate sleep, and stress and anxiety [7]. Among young people, musculoskeletal development and their tendency to use smartphones for texting and gaming are important factors due to increased repetitive exposures. The repetitive movements, poor posture, and prolonged use of smartphones for texting or playing games can lead to injuries in the fingers, hands, wrists, arms, elbows, shoulders, and neck [8, 9]. There was a significant difference between addicts and non-addicts and those without smartphone addiction in cervical spine repositioning errors. The study determined that individuals with severe smartphone addiction displayed the following postural changes: flexion, extension, right lateral flexion and left lateral flexion. These results were caused by errors in cervical repositioning. [10]. Using a smartphone requires the user to keep their head down and hold their device in front of them for extended periods of time. This position causes the head to tilt forward and the cervical lordotic curve to flatten. As a result, neck pain and a forward head posture may result [11, 12].

A myofascial trigger point (MTrP) occurs when a taut band of skeletal muscle is compressed and palpated that produces a typical pattern of pain. It is important to note that occult myofascial trigger points (MTrPs) do not spontaneously cause pain, but they can result in a limitation in range of motion and muscle weakness [13, 14]. It is believed that as stress increases, muscle fatigue occurs, which makes it more susceptible to the activation of additional trigger points [15–17] The prevalence of myofascial chronic pain and trigger points is up to 85%, and the incidence varies according to age and gender [18]. Although myofascial pain syndrome is common, its pathophysiological mechanisms have not been fully elucidated. However, trigger points are believed to arise from several categories, including muscle overuse, muscle trauma, psychological stress, or ergonomic, structural, or systemic factors. Myofascial trigger points in the cervical muscles may develop as a result of suboptimal postures, such as bending the neck downward while using a smartphone [1]. There is evidence in the literature that smartphone addiction is associated with musculoskeletal problems and trigger points resulting from long-term poor posture [15–17].

A MTrP is classified as active or latent. Both active and latent MTrPs can cause local and referred pain; however, active MTrPs can also produce patient symptoms, whereas latent MTrPs do not. It is possible for latent MTrPs to become active later [19, 20]. Thus, it has been suggested in the literature that identifying and treating latent trigger points can improve motor functions by reducing pain sensitivity and thus preventing the activation of latent trigger points, thereby contributing to the prevention of myofascial pain syndrome [20]. There are very few studies in the literature that have examined the relationship between smartphone addiction and trigger points, but they generally focus on the upper trapezius and sternocleidomastoid muscles [20–22]. In order to better understand and prevent the potential health risks associated with smartphone addiction, it is important to examine the presence of trigger points in the cervical, paraspinal, and scapular muscles, as well as the relationship between posture and musculoskeletal complaints among university students. The purpose of this study is to evaluate the relationship between smartphone addiction and trigger points in the cervical, paraspinal, and scapular regions, posture, and musculoskeletal complaints in university students.

## Methods

### Study design

This study was conducted to clarify the relationship between smartphone addiction and trigger points among university students at Istanbul *(Blinded) University between December 2018 and October 2019. An ethical committee permission numbered 29.11.2018/41 was obtained from Marmara University's Faculty of Health Science's Scientific Research Ethical Committee.

In order to determine the sample size, the effect size was calculated using trigger point data of the upper trapezius muscle obtained from a study by Alaca et al., which examined the extent to which trigger points existed between internet addicts and non-internet addicts [21]. According to the calculated effect size, it was determined that at least 110 participants would be required for 95% power (d = 0.63, α: 0.05). The number of participants was increased by 25% to prevent data loss.

**Fig. 1:**
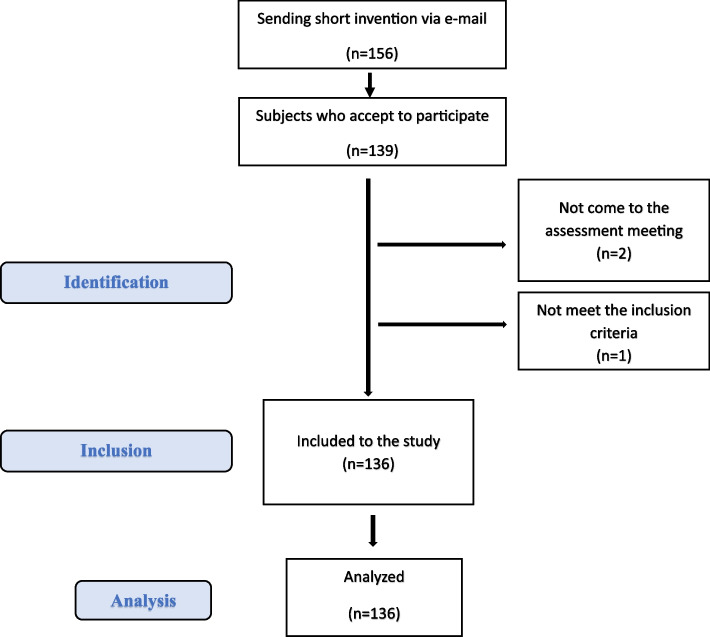
STROBE flow diagram

In total, 156 students were invited to participate in the study via e-mail. 139 of these students accepted and met face-to-face with ÖY researcher. Individually, two participants didn’t come to their appointment, 137 participants signed consent forms at their appointments, and then their eligibility for participation in the study was determined. There was one student who was excluded from the study due to non-compliance with the criteria. A survey and evaluation of each participant was conducted under the supervision of ÖY. As a result, there was no loss of data (Fig. 1).

The inclusion criteria was aged 18 and 25 participated who used smartphones and volunteered to participate.

The exclusion criteria included a history of neck trauma within the last six months, the treatment of trigger points within the last three months or a history of neck surgery.

In this study, in which we identified whether the participants is addicted to smartphones or not and then determine whether they have TrPs or not and TrPs and the other assessments then determine whether there is an association between smartphone addiction.

### Data collection

The informed consent was signed and participants were asked about their age, gender, dominant side, daily smartphone usage time, purpose, and posture. We asked the participants to select the most appropriate posture.

Obtained the daily time of smartphone usage by asking them how many hours are in a day. On the assessment form, they respond to the question.

Among the usage purposes asked for were social media, phone calls, messaging, and games. Their choice was based on which they use the most. [23].

**Fig. 2:**
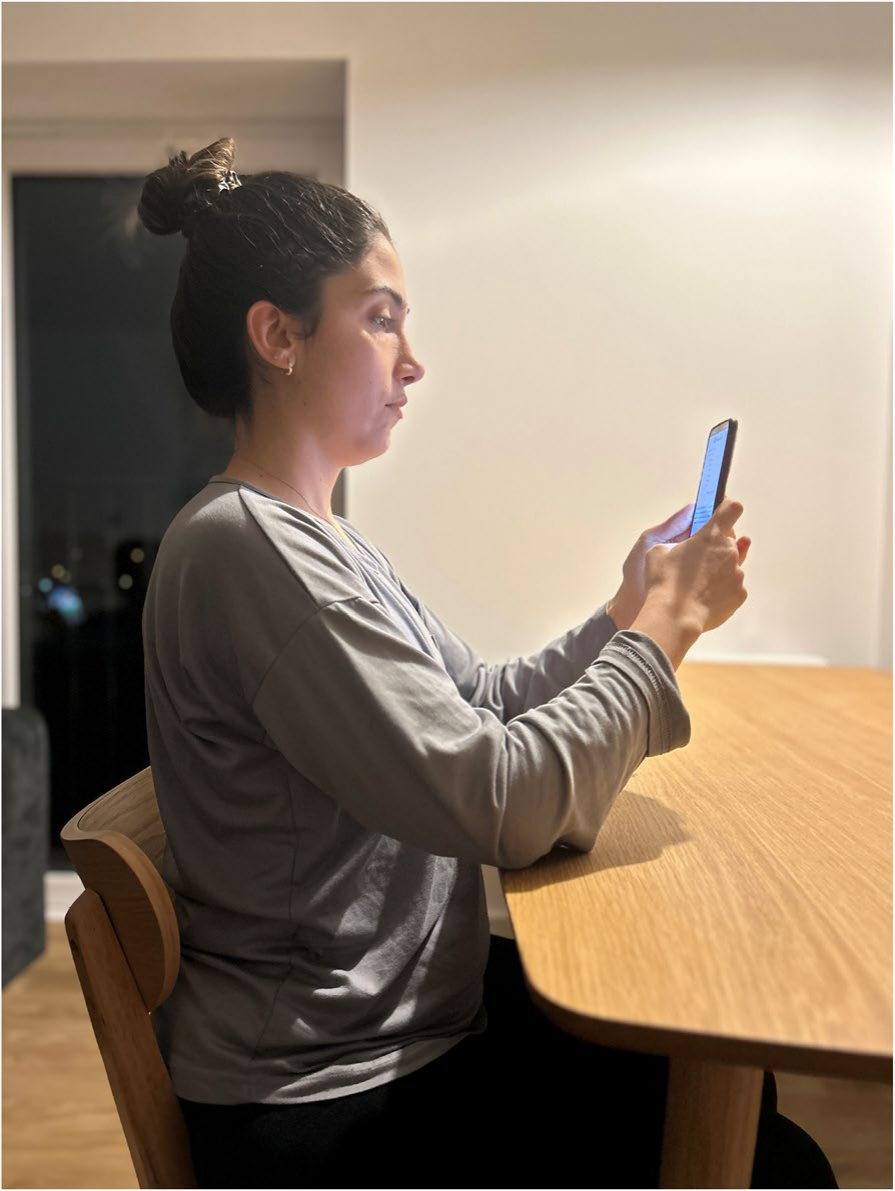
Neutral posture

**Fig. 3:**
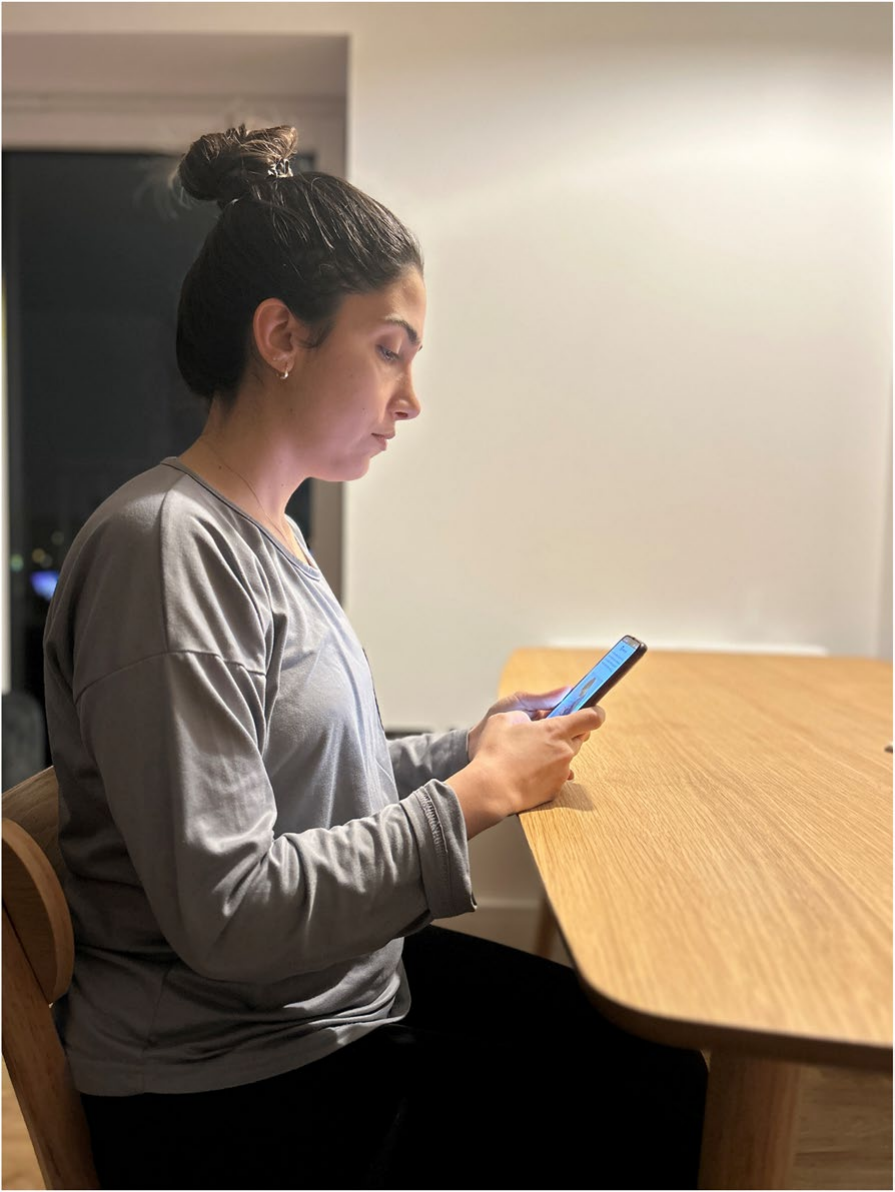
Middle bending posture

**Fig. 4:**
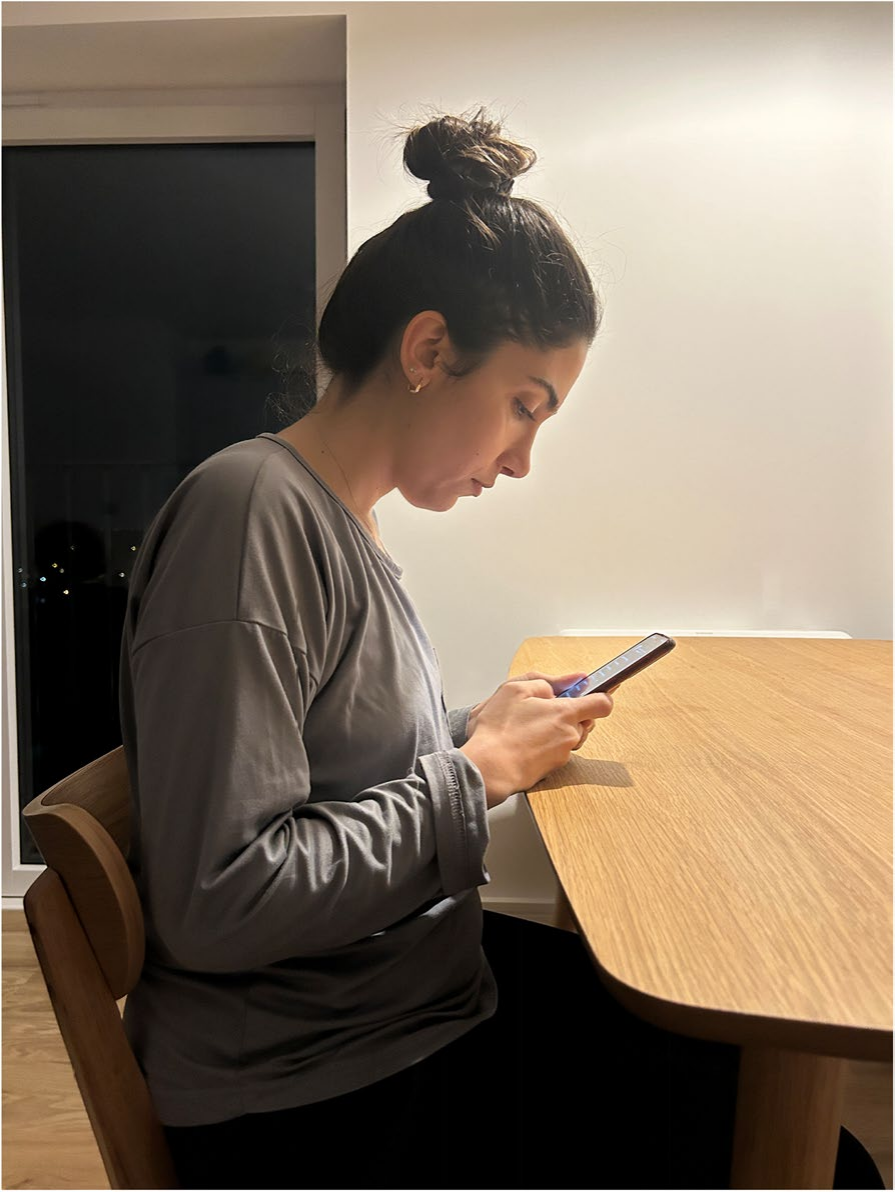
Maximum bending posture

In order to obtain information about the participants' smartphone usage posture, we showed them a photo of us while using the smartphone in the three general preferred postures (Neutral posture, middle bending posture, maximum bending posture). They selected one of them [12] (Figs. 2,3 and 4).

The Smartphone Addictive Scale-Short Form (SAS-SF) developed by Kwon et al. in 2013 was used to evaluate smartphone addiction [5]. In males, the cut-off value for SAS-SF is 31, the sensitivity is 0.867, and the specificity is 0.893; however, in females, the cut-off value is 33, the sensitivity is 0.875, and the specificity is 0.886 [24]. The Turkish translation of the SAS-SF is a valid, reliable, and consistent instrument. The cronbach alpha of SAS-SF is 0.947[24]. SAS-SF was administered to all participants. Smartphone addiction was determined by the scores the participants received from the scale.

The New York Posture Rating Chart (NYPRC) was used to evaluate the posture of the participants. The objective of this method is to observe and score any postural changes that may occur in 13 different parts of the body (head, neck, shoulder, scapula, upper thoracic, waist, rips, abdomen, hips, knees, legs, feet, and toes). There were three levels of scoring for each body segment: 5 (correct posture), 3 (slight deviation), and 1 (pronounced deviation). The higher the points, the better the alignment of the postural muscles [25].

Myofascial trigger points were questioned by Simon and Travel's criteria in the sternocleidomastoid, upper trapezius, levator scapula, rhomboid, infraspinatus and cervical extensor muscles. The criteria were as follows: -A tender spot in a taut band or nodule of skeletal muscle; -Subject recognition of pain when palpated; -Subject referred pain pattern; -Presence of a local twitch response [26].

Measurement of the tragus to wall distance was used to evaluate participants' forward head postures. In this study, participants were assumed to have a forward head with a tragus-to-wall distance of at least 10 cm [27, 28].

### Statistical analysis

Data were classified using qualitative and quantitative statistical methods using the SPSS 22.0 statistical program at a 95% confidence interval, and significance was determined at the *p* < 0.05 level. Kolmogorov-Smirnow test and histogram graphs were used to evaluate the variables' conformity to the normal distribution. For comparison analyses of quantitative variables between smartphone addicts and non-smartphone addicts, the one-sided Student’s t-test was used for those with normal distribution, and the one sided Mann–Whitney U test was used for those without normal distribution. The Pearson chi-square test was used in the analysis of categorical qualitative variables. Using the demographic information determined by the measurement, the mean, standard deviation, and minimum and maximum values were calculated.

## Results

In the end, 136 participants completed the study, 84 female and 52 male. In Table 1, participants' age, gender, dominant side, daily smartphone usage time, smartphone use purpose, presence of forward head posture, trigger points, SAS-SF score, New York posture rating chart score, wall to tragus distance, and smartphone usage posture information are presented.

**Table 1:** Descriptive statistics

	**Smartphone Addicts** ***n* = 61(44. 9)**		**Non-Smartphone Addicts** ***n* = 75 (55. 1)**		**p**
**Age (years)** *Mean* ± *SD*	20.05 ± 1.44		19.92 ± 1.49		0.638***
**Gender** *(N)*	Female	37	Female	47	0.810**
	Male	24	Male	28	
**Dominant Side** *(N)*	Right	60	Right	75	
	Left	1	Left	0	
**Daily time of smartphone usage (hours)** *Mean* ± *SD*	4.88 ± 0.85		3.01 ± 0.86		0.001***
**Smartphone usage purpose** *(N)*	Social media	39	Social media	49	0.128**
	Phone calls	3	Phone calls	8	
	Messaging	3	Messaging	8	
	Games	16	Games	10	
**Smartphone Addictive Scale—Short Form (score)**	40.34 ± 7.04		23.05 ± 5.77		
	39 (31–60)		24 (10–32)		
**Forward head posture**	Yes	54	Yes	60	0.179**
*(N)*	No	7	No	15	
**Totally number of trigger points** *Median (Min–Max)*	4 (0–9)		3 (0–7)		0.002*
**New York Posture Rating Chart (score)** *Median (Min–Max)*	59 (41–65)		59 (49–65)		0.819*
**Wall to tragus distance (cm.)** *Median (Min–Max)*	16 (12–26)		15 (11–26)		0.105*
**Smartphone usage posture** *(N)*	Posture-1:	50	Posture-1:	22	0.001**
	Posture-2:	11	Posture-2:	50	
	Posture-3:	0	Posture-3:	3	

Posture-1: Maximal bending posture; Posture-2: Middle bending posture; Posture-3: Neutral posture; min: minumum; max: maximum.;SD: standart deviation

^*^Mann–Whitney U test

^**^Pearson's chi-squared test

^***^Student’s t test

The age, gender, dominant side, smartphone usage purpose, forward head posture, New York posture rating score, and wall to tragus distance weren’t statistically different in addicts and non-addicts (*p* > 0.05).

A significant difference was found between the addicts and non-addicts in terms of the daily smartphone usage time, the total number of trigger points, and the smartphone usage postures. There was 61 (% 44.9) addictive and 75 (% 55.1) non-addictive participants according to the cut off value (33 for female, 31 for male) of SAS-SF score. Participants who is smartphone addicts, the daily time spent on smartphones and the total number of trigger points were greater than those in the non-addicts, and it was revealed that the maximal bending posture was favored most often (*p* < 0.05).

In the study, the trigger points in muscles were compared between the addicts and non-addicts. Based on Pearson chi-square testing, the smartphone addicts showed a statistically significant difference in the right levator scapula, left levator scapula, and right cervical extensor muscles (Table 2).

**Table 2:** Comparison of muscles with trigger point between participants

Trigger points in muscles	Smartphone Addicts *n* = 61		Non-Smartphone Addicts *n* = 75		p*
**Upper trapezius (Right)**	Yes	42	Yes	48	0.552
	No	19	No	27	
**Upper trapezius (Left)**	Yes	11	Yes	14	0.924
	No	50	No	61	
**SCM (Right)**	Yes	20	Yes	14	0.059
	No	41	No	61	
**SCM (Left)**	Yes	7	Yes	3	0.097
	No	54	No	72	
**Levator scapula (Right)**	Yes	13	Yes	5	**0.012**
	No	48	No	70	
**Levator scapula (Left)**	Yes	7	Yes	2	**0.040**
	No	54	No	73	
**Infraspinatus (Right)**	Yes	20	Yes	16	0.132
	No	41	No	59	
**Infraspinatus (Left)**	Yes	2	Yes	6	0.245
	No	59	No	69	
**Rhomboid (Right)**	Yes	7	Yes	9	0.925
	No	54	No	66	
**Rhomboid (Left)**	Yes	2	Yes	3	0.114
	No	59	No	72	
**Cervical erector muscles (Right)**	Yes	20	Yes	12	**0.022**
	No	41	No	63	
**Cervical erector muscles (Left)**	Yes	13	Yes	9	0.142
	No	48	No	66	

^*^Pearson's chi-squared test

## Discussion

In this study investigating the relationship between smartphone addiction and trigger point in university students, it was found that smartphone addiction was associated with postural changes and myofascial trigger points. Since smartphones are being used more frequently by young people, that smartphone use reduces social interaction, causes insomnia, and contributes to a number of musculoskeletal problems there should be a growing awareness of addiction [29]. The excessive and compulsive use of smartphones may result in myofascial trigger points and subsequent postural changes. Smartphone usage is often associated with suboptimal postures, such as hunching over screens or craning the neck downward. As a result, myofascial trigger points can develop in the muscles, causing tenderness and pain. In this context, the purpose of this study was to investigate the relationship between smartphone addiction and trigger points and posture in university students. Several articles have been published in recent years that examine the relationship between smartphone addiction and health outcomes among university students. It is stated in the studies that university students are at a high risk for smartphone addiction. [30, 31]. The difficulties of academic life, fears and concerns about the future may contribute to the high stress levels of university students, and because of these high stress levels, they are more inclined to use smartphones [32, 33]. We therefore selected a sample of university students for our study. As part of this study, it was observed that as smartphone addiction increased, the number of trigger points increased, and forward head posture became more prevalent. There are a few studies in the literature that investigate the relationship between trigger points and smartphone usage. The trigger points in the hand muscles of smartphone users were investigated by Mehta & Vijayakumar in 2020 [20]. The relationship between internet addiction and trigger points in the upper trapezius muscle was examined by Alaca et al. in 2020 [21]. However, in this study, unlike the study of Alaca et al., the relationship between addiction in smartphone users and trigger points in the neck and surrounding muscles was examined, including upper trapezius, levator scapula, rhomboid, SCM, infraspinatus and cervical erector, along with postures of smartphone users.

The myofascial trigger point (MTP) is a hyperirritable area within a taut band of skeletal muscle that is painful when compressed [9]. Trigger points can be either active or latent. The symptoms of both active and latent trigger points include referred pain, tenderness, and autonomic dysfunction. When a MTP is active, referred pain can occur spontaneously, while when a MTP is latent, referred pain can only occur when a manual compression is applied [10]. In this study, the presence of latent trigger points in smartphone users was investigated. As latent trigger points may become active later [19], early examination may be beneficial in preventing musculoskeletal complaints. In the literature, some hypotheses have been proposed to explain the etiology of latent trigger points. Among these factors are sustained low-level muscle contractions, uneven intramuscular pressure distribution, direct trauma, eccentric contractions in muscles with deconditioning, and maximal or submaximal muscle contractions [34, 35]. According to our study, the smartphone-addicted participants had more latent trigger points than the non-addicted participants. Furthermore, smartphone use time was significantly greater in the smartphone addicts compared to the non-addicts on a daily basis. A prolonged use of smartphones places continued mechanical stress on the tendons, muscles, and surrounding tissues, resulting in an increase in flexion of the cervical spine and a change in posture [34]. According to Jung et al. in 2016, people with forward head posture (FHP) are very likely to have altered craniovertebral angle and scapular index after using smartphones for more than 4 h in a day [35]. As the craniovertebral angle indicates head on trunk positioning, FHP is a common postural disorder. As a result of the forward head posture, the cervical extensors and SCM muscles shorten. In this study, a majority of smartphone users exhibited a forward head posture. University students who held their heads in a forward-leaning position during school exams and those who had a forward head posture were found to have numerous trigger points in the levator scapula and upper trapezius muscle [36]. As a result of this study, more trigger points were found in the levator scapula and cervical extensor muscles of smartphone addicts. As well as the fact that the prevalence of trigger points in the levator scapula muscle is very high [37], it has also been associated with cervical postural disorders and myofascial pain syndromes [38, 39] The presence of a trigger point can cause muscle tightness and shortening and put pressure on the points where the muscles connect to the bones [39]. Because the levator scapula attaches to both the cervical vertebrae and the scapula, it plays an important role in both shoulder and cervical biomechanics [37, 40]. Releasing the tightness of the levator scapula muscle has found to be effective in improving shoulder proprioception and correcting forward head posture [40]. Smartphone use may cause postural changes and increase the degree of flexion in the cervical and the upper thoracic spine [41]. In this study, the most preferred postures of individuals using smartphones were questioned and the presence of trigger points was investigated. In smartphone-addicted participants, both the number of trigger points and the presence of trigger points in the levator scapula muscle were significantly higher than in those who were not smartphone-addicted. Choi et al. examined the three most preferred postures among smartphone users [42]. In this study, participants were asked to choose their most preferred phone usage postures among these three postures [42]. Among the smartphone-addicted participants, the maximum forward bending posture was more preferred than among the non-addicted participants. In the maximum forward bending posture, the degree of flexion of the cervical was more pronounced than in the other two postures. In the maximum bending posture, the cervical region was in total flexion. The upper cervical and total cervical flexion angles of computer workers with pain in the upper trapezium and those with pain in the levator scapula muscle were compared in a study. Patients with pain in the levator scapula muscle had a lower upper cervical flexion angle and a higher total cervical flexion angle than patients who suffered from pain in the upper trapezius muscle [43]. The data from this study also supports Yoon's study. This is because, in this study, the maximum bending posture in which the cervical region was totally flexion was higher in smartphone addicts, and the trigger point in the levator muscle was higher than in non-addicted participants. Statistically, smartphone addicts had a higher number of trigger points than non-addicts in this study. We consider that poor posture is the major reason for the high number of trigger points in the smartphone addicts. Using surface EMG, Choi et al. in 2016 investigated muscle fatigue in three different postures (maximal bending, mild bending, and neutral posture) in university students [42]. As compared to the middle bending posture, the maximum bending posture resulted in more fatigue in the cervical muscles. Myofascial trigger points are caused by prolonged poor posture, low-level muscle contractions, and muscle fatigue [34, 44]. As a result of these data in the literature, we believe that more trigger points are found in the smartphone addicted participants in our study as a result of these factors.

Chronic musculoskeletal pain is considered a biopsychosocial condition in which biological factors, as well as contextual, cognitive and emotional factors, significantly affect the perception of pain. It has been reported that pain focus, disease perception, self-efficacy, and fear avoidance behaviors are effective in pain management. [45] In this context, it can be thought that there are many factors affecting myofascial pain and trigger points seen in smartphone addicts and university students. It has been found that university students who are internet addicts have more physical fatigue than those who are not university students [46]. Another study reported a correlation between internet addiction and musculoskeletal pain severity [5]. Studies conducted on university students show a negative correlation between phone addiction and exercise intensity [6]. The results of a systematic review conducted in 2022 revealed that exercise and psychological interventions can help reduce smartphone addiction. [7] This study aimed to reveal the alarming increase in smartphone addiction among university students and its possible musculoskeletal negative consequences. In the literature, studies examining the relationship between smartphone addiction and trigger points have mostly focused on upper trapezius and SCM. However, in this study, we evaluated muscle groups such as infraspinatus, rhomboid and cervical erectors in addition to upper trapezius and SCM in terms of the presence of myofascial trigger points. While this is the strength of our study, there are also some limitations. One of the limitations of our study is that other conditions that may be associated with smartphone addiction, such as exercise habits, nicotine, computers, and alcohol, were not evaluated. We believe that future multicenter studies may shed light on addiction and related conditions. Another limitation is that some evaluation parameters were self-reported during the data collection phase, and this may have led to different types of bias. Although palpation is considered the gold standard when performed by an experienced clinician, trigger point evaluation could be performed with a more objective method.

## Conclusions

As a result, we found that smartphone addiction, which has been associated with long-term usage in university students, can be associated with postural changes and trigger points in the bilateral levator scapula and right cervical erector muscles. This situation may pose a threat to the health of the musculoskeletal system. It would be appropriate to consider the biopsychosocial model when assessing the relationship between smartphone addiction and physical health in future studies. It is recommended that public health programs should be developed to raise awareness about the negative effects of smartphone addiction, encourage screen breaks, and emphasize the importance of physical activity and exercise on a regular basis.

## Data Availability

The datasets used and/or analysed during the current study available from the corresponding author on reasonable request.
